# Biases and corrections in total factor productivity evaluation in Chinese public hospitals: an opinion under the policy lens of new quality productive forces

**DOI:** 10.3389/fpubh.2026.1892483

**Published:** 2026-10-05

**Authors:** Xuesong Li

**Affiliations:** School of Economics and Management, Taizhou University, Taizhou, Jiangsu, China

**Keywords:** digital governance, new quality productive forces, public hospitals, public-welfare orientation, total factor productivity

## 1 Introduction

Health systems face rising demand, constrained resources, and pressure for fiscal sustainability. Productivity is therefore central to health policy, but hospital performance cannot be interpreted apart from quality, outcomes, and equity. The World Health Organization defines quality through effectiveness, safety, people-centredness, timeliness, equity, integration, and efficiency (1). A recent scoping review likewise found that healthcare efficiency is often narrowly specified around technical or productive efficiency, with limited inclusion of care quality and health outcomes (2).

In hospitals, total factor productivity (TFP) is the change over time in the relationship between a bundle of outputs and a bundle of labor, capital, and intermediate inputs. Data envelopment analysis (DEA)-Malmquist studies commonly decompose TFP change into efficiency change and technological change. TFP is thus an estimated input-output performance construct, not a count of technology, investment, or service activity. Chinese studies report heterogeneous productivity patterns across Beijing public hospitals, provincial health systems, and tertiary public hospitals, and recent work shows that estimates can change when quality, safety, environmental conditions, and undesirable outputs are incorporated (3–6).

China's public hospital reforms have shifted from resource expansion and service-volume growth toward quality, operational efficiency, sustainability, patient experience, and public-welfare orientation (7–9). New quality productive forces (NQPF) emphasize technological breakthroughs, innovative factor allocation, and TFP improvement as features of productivity upgrading (10, 11). However, NQPF should complement rather than replace established frameworks. Quality-adjusted productivity modifies measured output to reflect complexity and care results; value-based healthcare defines the normative objective as better patient outcomes relative to the resources used (12). NQPF adds a Chinese policy and governance lens: it asks whether technology, data, workforce capability, organizational redesign, and incentive reform reconfigure production factors and move the hospital's effective production frontier under public-welfare constraints.

This Opinion uses ‘bias' in two related senses. Model-specification bias arises when formal TFP models rely on activity outputs while omitting quality or undesirable outputs. Interpretation and performance-assessment bias arises when service volume, cost reduction, or digital investment is treated as sufficient evidence of productivity improvement. Digital investment is an input or enabling condition, not a direct proxy for TFP. The article identifies these biases and proposes a quality-inclusive approach to TFP evaluation in Chinese public hospitals.

## 2 Two biases in TFP evaluation under an NQPF-informed lens

### 2.1 Measurement and interpretation bias: conflating activity, cost containment, and meaningful output

Many hospital TFP applications use visits, discharges, operations, or bed-days as outputs. These are legitimate activity measures, but without case-mix, risk, or quality adjustment they may reward low-value volume. Duplicative testing, avoidable admissions, or inappropriate procedures can increase measured output without improving health. Chinese evidence using medical-quality constraints in county-level public hospitals found that efficiency estimates differed when quality was incorporated (13). Studies using undesirable outputs similarly show that omitting adverse outcomes can conceal quality losses (6, 14).

Cost containment is not itself TFP improvement. It affects measured productivity only through the specified inputs and outputs, and its policy meaning also depends on quality, access, and patient burden. Chinese policy prohibits linking departmental or clinician remuneration to revenue from medicines, consumables, or tests (15). After drug markups were abolished, drug expenditure fell but induced demand for other medical services increased (16). Thus, volume growth or lower expenditure should not be interpreted as socially meaningful productivity gain unless quality, safety, access, and resource use improve together.

### 2.2 Interpretation bias: treating digital investment as evidence of productivity improvement

Digital platforms, information systems, and equipment are capital inputs or enabling infrastructure. They do not directly measure TFP, and their benefits may appear only after implementation costs and learning lags. Digitalization can contribute to TFP when it reduces wasteful inputs or increases quality-adjusted output through pathway redesign, rule harmonization, interoperability, and clinical or managerial decision support (17–20).

Productivity conversion depends on organizational capability, staff digital competence, clinical-administrative coordination, and data governance (17, 20). Weak interoperability can delay communication, encourage duplicative testing, and prolong visits or stays (19). Accordingly, system counts, investment amounts, or digital-maturity scores should be treated as process measures or explanatory variables in second-stage analysis, not as substitutes for TFP. The relevant question is whether digitalization changes the input-output transformation of care.

## 3 Correcting TFP evaluation toward quality-inclusive productivity

### 3.1 Indicator architecture for quality-inclusive TFP

A quality-inclusive framework should distinguish four indicator groups because each performs a different analytical function (Table 1). Case-mix and risk-adjustment indicators normalize differences in expected resource needs or outcomes; they are not achievements in themselves. Quality and outcome indicators represent care results. Undesirable outputs capture harms or waste that should be minimized. Public-welfare and equity indicators assess social obligations that activity-based output does not capture (6, 13, 14).

**Table 1:** Proposed indicator groups and their roles in productivity evaluation.

Indicator group	Examples	Role in productivity evaluation
**Case-mix and risk adjustment**	Case-mix index (CMI); diagnosis-related group (DRG) or diagnosis-intervention packet (DIP) weights; age, comorbidity, severity, emergency status	Normalize expected resource use and outcomes; weight activity outputs or stratify peer groups. These variables are not outputs by themselves.
**Quality and outcomes**	Risk-adjusted mortality; complications; hospital-acquired infection; readmission; patient-reported outcome measures (PROMs); patient experience	Use as quality weights, desirable outcomes, or minimum quality constraints after risk adjustment.
**Undesirable outputs and waste**	Avoidable readmissions; preventable safety events; infections or complications if not already modeled above; duplicative tests; low-value care; unwarranted expenditure	Model as bad outputs in directional-distance/SBM frameworks or as pre-specified penalties; avoid double counting.
**Public-welfare and equity**	Access for vulnerable or rural patients; financial protection; complex and critical care; emergency and public-health functions; support to primary care and regional coordination	Treat as additional outputs, non-discretionary constraints, or a parallel scorecard; do not reduce them to case-mix controls.

CMI, case-mix index; DRG, diagnosis-related group; DIP, diagnosis-intervention packet; PROMs, patient-reported outcome measures; SBM, slacks-based measure.

Boundaries must be specified before estimation. Readmissions, infections, and complications may be used either as quality outcomes or as undesirable outputs, but the same indicator should not enter twice. Equity variables should not be absorbed solely into risk adjustment, because doing so may hide the hospital's public-welfare contribution.

### 3.2 Digital transformation as a determinant of productivity conversion

Digital evaluation can use three process domains. Workflow-efficiency indicators include appointment waiting time, test turnaround time, discharge settlement time, repeated data entry, and staff documentation burden. Coordination indicators include electronic health record (EHR) interoperability, test-result sharing, cross-departmental exchange, and bed-allocation response time. Quality-support indicators include the effectiveness of clinical alerts, medication-safety prompts, abnormal-expenditure warnings, and patient-safety tracking.

These indicators identify mechanisms through which digitalization may influence productivity; they are not TFP outputs by default. They can be examined as intermediate outcomes, implementation covariates, or second-stage determinants, while the primary productivity model tests whether inputs fall or quality-adjusted outputs rise.

### 3.3 Incorporating the indicators into productivity evaluation

Implementation can proceed in stages. First, estimate conventional TFP with transparent labor, capital, intermediate-input, and activity-output definitions. Second, apply case-mix or risk weights and add selected quality outcomes. Third, model harms through directional-distance or slacks-based measure (SBM) DEA methods, or through pre-specified penalties when data are insufficient for frontier models. Public-welfare and equity indicators may be included as additional outputs, non-discretionary constraints, or a companion scorecard rather than forced into one composite index.

Conventional and quality-inclusive TFP estimates should be reported side by side, with decompositions and sensitivity analyses across indicator sets, weights, and peer groups. Digital indicators should enter as explanatory or mediating variables. Payment, fiscal compensation, and internal performance systems can then reward sustained improvement in quality-inclusive productivity while monitoring for under-provision, selective admission, cost shifting, and gaming.

## 4 Discussion

The central problem is therefore both measurement-related and interpretive. Activity growth, digital investment, and cost containment are not independently sufficient evidence of productivity improvement. Quality-adjusted productivity provides the measurement correction, value-based healthcare provides the outcome-and-resource objective, and NQPF adds attention to factor reconfiguration, organizational capability, and institutional incentives under public-welfare constraints.

Policy alignment is essential. Fiscal compensation should recognize public health functions, complex and critical care, regional coordination, primary-care support, and emergency preparedness. Insurance payment should combine cost control with safeguards for quality, readmissions, complications, access, and patient financial burden. Hospital assessment and remuneration should reduce dependence on revenue and workload and give greater weight to outcomes, patient experience, low-value care reduction, equity, and public-welfare contribution (7–9, 15, 16).

Future research should develop and empirically validate indicator sets suited to different tiers of Chinese public hospitals. Reporting conventional and quality-inclusive TFP together would make the source of any divergence visible and help translate NQPF from a broad policy concept into testable governance performance.

### 4.1 Limitations

This Opinion is conceptual and presents no original empirical validation. Patient-level severity, outcome, cost, digital, and equity data are not uniformly available, and coding practices, diagnosis-related group (DRG) or diagnosis-intervention packet (DIP) rules, and hospital functions vary across regions and levels. Indicator overlap creates double-counting risks, while weights, frontier specifications, and peer-group definitions affect comparability. Digital effects may also be delayed and endogenous. Future studies should validate indicator reliability, harmonize definitions, test alternative specifications, examine unintended incentives, and assess whether quality-inclusive TFP predicts meaningful improvements in patient and public value.
